# NLRP3-inflammasome inhibition prevents high fat and high sugar diets-induced heart damage through autophagy induction

**DOI:** 10.18632/oncotarget.20763

**Published:** 2017-09-08

**Authors:** Luís E. Pavillard, Diego Cañadas-Lozano, Elísabet Alcocer-Gómez, Fabiola Marín-Aguilar, Sheila Pereira, Avril A.B. Robertson, Jordi Muntané, Bernhard Ryffel, Matthew A. Cooper, José L. Quiles, Pedro Bullón, Jesús Ruiz-Cabello, Mario D. Cordero

**Affiliations:** ^1^ Research Laboratory, Oral Medicine Department, University of Sevilla, Sevilla, Spain; ^2^ Institute of Biomedicine of Seville (IBiS), “Virgen del Rocío” University Hospital, IBiS, CSIC, University of Seville, Seville, Spain; ^3^ Institute for Molecular Bioscience, The University of Queensland, Brisbane, Australia; ^4^ Department of General Surgery, Hospital Universitario Virgen del Rocio, CSIC, Universidad de Sevilla, Sevilla, Spain; ^5^ University and CNRS, UMR7355, Orléans, France; ^6^ CIBER de Enfermedades Respiratorias, Madrid, Spain; Advanced Imaging Unit, Centro Nacional de Investigaciones Cardiovasculares, and Universidad Complutense Madrid, Madrid, Spain; ^7^ Centro de Investigación Biomédica en Red de Enfermedades Hepáticas y Digestivas (CIBEREHD o Ciberehd), Instituto de Salud Carlos III, Madrid, Spain; ^8^ Institute of Nutrition and Food Technology “José Mataix Verdú”, Department of Physiology, Biomedical Research Center, University of Granada, Granada, Spain

**Keywords:** NLRP3-inflammasome, cardiac damage, autophagy, MCC950

## Abstract

The NLRP3-inflammasome complex has emerged as an important component of inflammatory processes in metabolic dysfunction induced by high-caloric diets. In this study, we investigate the molecular mechanisms by which NLRP3 inhibition may attenuate diet-induced cardiac injury. Here we show the cardiac damage induced by high sugar diet (HSD), high fat diet (HFD) or high sugar/fat diet (HSFD) over 15 weeks. Genetic ablation of NLRP3 protected against this damage by autophagy induction and apoptotic control. Furthermore, NLRP3 inhibition by the selective small molecule MCC950 resulted in similar autophagy induction and apoptotic control in hearts after diets. These data were reproduced in THP-1 cells treated with MCC950 and cultured in media supplemented with serum from mice dosed with MCC950 and fed with diets. NLRP3 inhibition exerted beneficial metabolic, and autophagic adaptations in hearts from obesogenic diets. The inhibition of NLRP3 activation may hold promise in the treatment of metabolic and cardiovascular diseases.

## INTRODUCTION

Cardiovascular disease (CVD) is the leading cause of death in the world with a high prevalence both in industrialized but also in low- and middle-income countries [1]. Studies show that only about 8% US adults had a low risk profile for cardiovascular disease between 1999 and 2004 [2]. In Spain, the level of ideal cardiovascular health is as low as in the United States [3]. Energy-rich diets high in fat, processed and refined carbohydrate foods, together with sedentary lifestyles have led to an increased CVD risk, and this effect is thought to be mediated primarily by elevated LDL cholesterol blood levels. In these cases, the role of obesity is especially relevant because multifactorial pathophysiological mechanisms interrelate this condition with other diseases such as type 2 diabetes, renal disease, and metabolic syndrome. All these obesity-induced complications share a low-grade systemic inflammatory response characterized by upregulated cytokine production and activated signaling pathways [4], yet the specific inflammation-related signaling pathways contributing to the metabolic dysfunction are still unknown. On this respect, the NLRP3-inflammasome complex has been described as a ‘danger sensor’, which triggers an innate immune response to a multitude of endogenous metabolic danger patterns inducing sterile inflammation [5]. NLRP3 activation induces the recruitment and autocatalytic activation of the cystein protease caspase-1, which processes the cytosolic precursors of the related cytokines IL-1β and IL-18, and leads to the secretion of the biologically active form of these cytokines [6].

Mice fed with HFD developed NLRP3-inflammasome activation in adipose, renal and hepatic tissues [7–9]. Interestingly, the absence of NLRP3 in these animals protected them to the adverse effects of HFD and the development of pathophysiology [7–9]. However, the role of NLRP3 in the pathogenesis of HFD-induced CVD appearance has not been characterized. Recent observations identified a specific role for NLRP3-inflammasome in the maladaptive response in cardiac tissue after ischemia/reperfusion in mice under high-fat high-fructose diets [10] and in the diabetic cardiomyopathy obseved after HFD and streptozotocin treatment in rats [11]. To understand the role of the NLRP3-inflammasome in diet induced-cardiac damage, we assesed the response of NLRP3 -/- mice, and the effect of the NLRP3 inhibitor MCC950 on high sugar diet (HSD), HFD and high sugar/fat diet (HSFD).

## RESULTS

### Absence of the NLRP3 protects from HSD, HFD and HSFD-induced obesity

**Figure 1:**
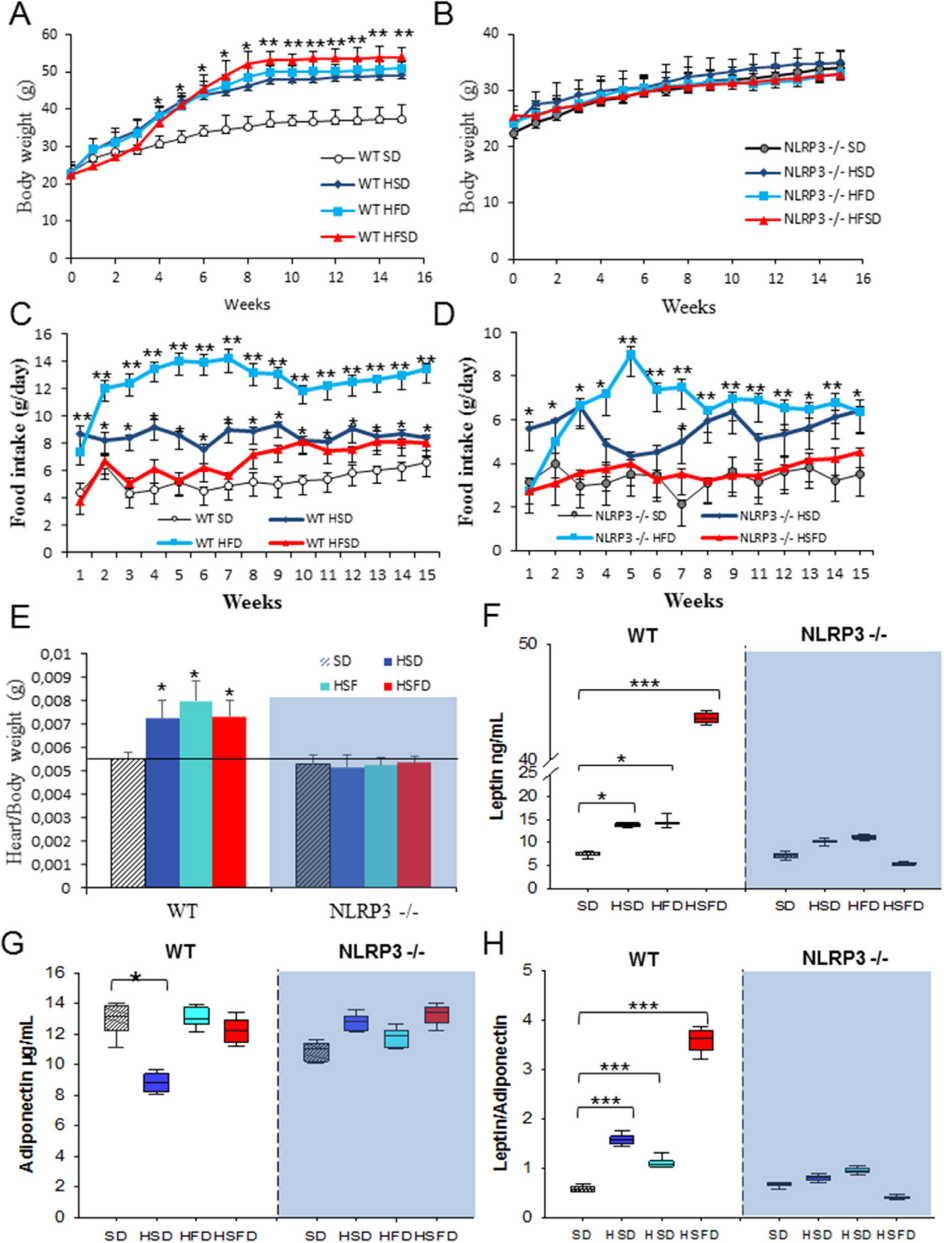
Nlrp3 signaling suppression prevents obesity induced effects of the HSD, HFD and HSFD diets **(A** and **B)** Body weight chart of diets-induced obesity in WT and NLRP3 -/- mice. The mice were fed with the diets for 15 weeks and their body weights were monitored weekly. **(C** and **D)** Average daily oral food intake normalized to body weight, measured on the different diets. The food intake of mice was measured was monitored weekly. **(E)** Heart weight normalized to body weight. **(F-H)** Levels of leptin, adiponectin, and ratio in plasma. Blood samples were collected after overnight fasting. All data are presented as means ± SEM, n = 10 mice; ^*^P < 0.05, ^*^P < 0.005, ^***^P < 0.001.

We have investigated the role of the NLRP3-inflammasome in mice lacking the NLRP3 gene and exposed to the different diets inducing obesity. Food intake and bodyweight evolution were examined weekly during 15 weeks in each group. The different diets have been shown to induce significant weight gain in WT mice (Figures 1A and 1B) [7] despite increased food consumption that was similar in all WT and NLRP3-/- mice groups (Figures 1C and 1D). Predictably, hearts from WT mice were substantially heavier than SD-fed animals at the end of the study (*p<0.05*) (Figure 1E and Supplementary Figure 1). This finding was not altered in the NLRP3 -/- genotype on any obesity-associated diet.

In humans, high-caloric diets cause numerous alterations including increased glucose, cholesterol levels and leptin/adiponectin dysregulation leading to cardiovascular disease, metabolic syndrome, and non-alcoholic fatty liver disease [12, 13]. The HSD, HFD and HSFD-fed WT mice showed increased serum levels of leptin (HSD and HFD, p<0.05; HSFD, p<0.001) and leptin/adiponectin ratio (p<0.001) (Figures 1F-1H), including abnormal serum levels of glucose, cholesterol, triglycerides, hepatic transaminases, uric acid and creatinine (Supplementary Table 2). These changes were not observed in NLRP3 -/- mice.

### NLRP3-deficiency induce a moderate protection against oxidative stress in heart and inflammation

**Figure 2:**
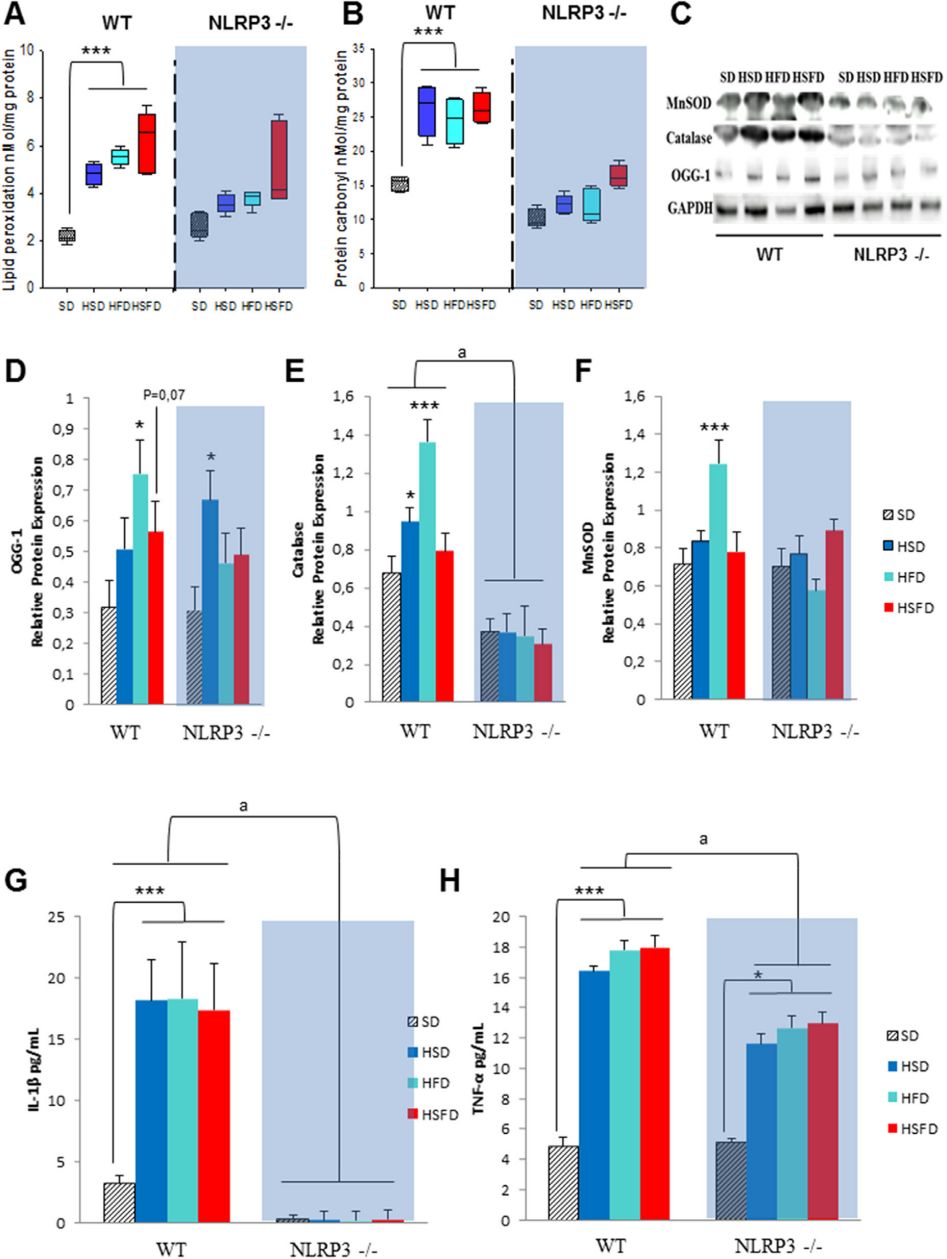
Nlrp3 signaling elimination protects against oxidative and inflammatory effects of the HSD, HFD and HSFD diets **(A** and **B)** Levels of lipid peroxidation, and protein carbonyls in heart tissues from mice. **(C)** Protein expression level of 8-oxoguanine glycosylase (OGG-1), MnSOD and catalase. **(D-F)** Densitometric quantification of protein expression levels of western blots in heart tissues from mice relative to GAPDH (loading control). All data are presented as means ± SEM with a representative blot, n = 6–10 mice. **(G** and **H)** IL-1β and TNF-α levels in serum from WT and NLRP3 -/- mice fed with the differents diets were determined by ELISA. All data are presented as means ± SEM with a representative blot, n = 10 mice; ^*^P < 0.05, ^***^P < 0.001 HSD, HFD and HSFD *vs* SD; ^a^P < 0.005 WT *vs* NLRP3 -/-.

Oxidative stress has an essential role in inflammasome activation [14], and has been implicated in the cardiac pathophysiology induced by obesogenic diets [15]. Accordingly, analysis of oxidative stress biomarkers in hearts of HSD, HFD and HSFD fed WT mice revealed significant increases in lipid peroxidation (Figure 2A), protein carbonyls (Figure 2B) and of 8-oxoguanine glycosylase (OGG1) (Figures 2C and 2D). Compared to WT mice, NLRP3 -/- mice had a trend toward an elevated lipid peroxidation and OGG-1 levels (Figures 2A-2D). This finding was consistent with the antioxidant biomarkers were not increased in these knockout animals challenged with obesogenic diets, suggesting perhaps the absence of compensatory defense mechanisms (Figures 2E and 2F) in this genotype. These results were also accompanied by elevated serum inflammatory biomarkers such as IL-1β and TNF-α in WT mice (Figures 2G and 2H). Interestingly, no differences in serum levels of IL-1β and a moderate increase in TNF-α were observed in all NLRP3 -/- groups (Figures 2G and 2H).

**Figure 3:**
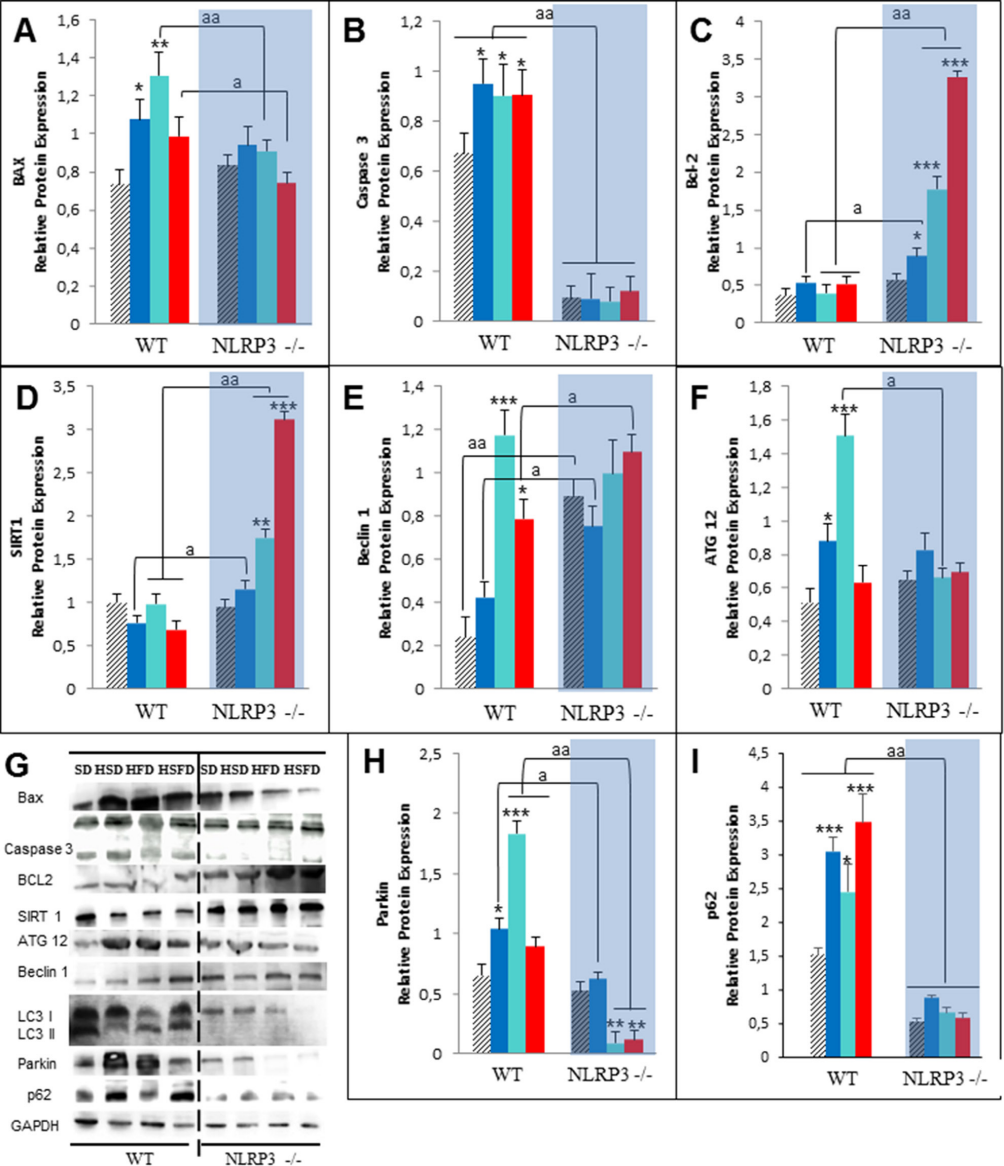
Changes in apoptosis and autophagy in response to HSD, HFD and HSFD diets in WT and NLRP3 -/- mice **(A-C)** Densitometric quantification of protein expression levels of Bax, Caspase 3 and Bcl-2 in heart tissues from mice relative to GAPDH (loading control). **(D)** Densitometric quantification of protein expression levels of Sirt1 in heart from mice. **(E-F)** Densitometric quantification of protein expression levels of autophagy markers in the heart. **(G)** Western blot analysis with representative blot including LC3. **(H** and **I)** Expression levels of Parkin and p62 level in the hearts of WT and NLRP3 -/- mice. All data are presented as means ± SEM, n =6–8 mice; ^*^P < 0.05, ^**^P < 0.005, ^***^P < 0.001 HSD, HFD and HSFD *vs* SD; ^a^P < 0.005 WT *vs* NLRP3 -/-.

Because high fat diet is also associated to cardiomyocite apoptosis, which is coupled with oxidative stress and inflammation dysregulation [16], we determined several apoptotic biomarkers. Here HSD, HFD and HSFD feeding resulted in a significant increase in protein expression of cardiac BAX and active caspase 3 (Figures 3A, 3B and 3G) accompanied by reduced cardiac expression levels of antiapoptotic protein Bcl-2 (Figures 3C and 3G) in WT mice. NLRP3 -/- mice showed reduced contents of apoptosis-related markers, consistent with the accumulation of Bcl-2 which stimulates antiapoptotic activity in heart (Figures 3C and 3G). Interestingly, these observations were also accompanied by increased SIRT1 protein expression levels in knockout mice (Figures 3D and 3G) which has a role in heart protection, for example, by anti-inflammatory and antioxidant effects [16].

### NLRP3-deficient mice show an autophagic protection against HSD, HFD and HSFD-Induced heart damage

One of the most important mechanisms in the cardiac protection/injury is autophagy, which is impaired during aging and diet-induced obesity, producing a less effective clearance process and contributing to important cardiac damage [17]. HSD, HFD and HSFD induced high expressions of two important autophagic proteins such as Beclin 1 and ATG-12 (Figures 3E, 3F and 3G) in WT animals. Furthermore, we observed an impaired autophagic flux determined by expression of LC3-II and increased expression of proteins involved in clearance pathways such as PARKIN and p62/SQSTM1 (Figures 3G, 3H and 3I). Interestingly, all NLRP3 -/- groups showed an increased and more efficient autophagic machinery (Figures 3E-3I). As LC3-II overexpression can evoke either an increment of autophagy or an impairment of the autophagic flux, these LC3-II changes must to be interpreted together with the p62/SQSTM1 levels and minding that LC3-II itself is subject to autophagic degradation at the lysosome [18]. As, NLRP3 -/- mice showed a more efficient autophagy induction, as the lower levels of LC3-II (Figure 3G) and the reduced levels of proteins such as parkin (Figure 3H) and p62/SQSTM1 (Figure 3I) is suggesting.

### Changes in gene expression of adhesion molecules and fibrosis markers in heart

**Figure 4:**
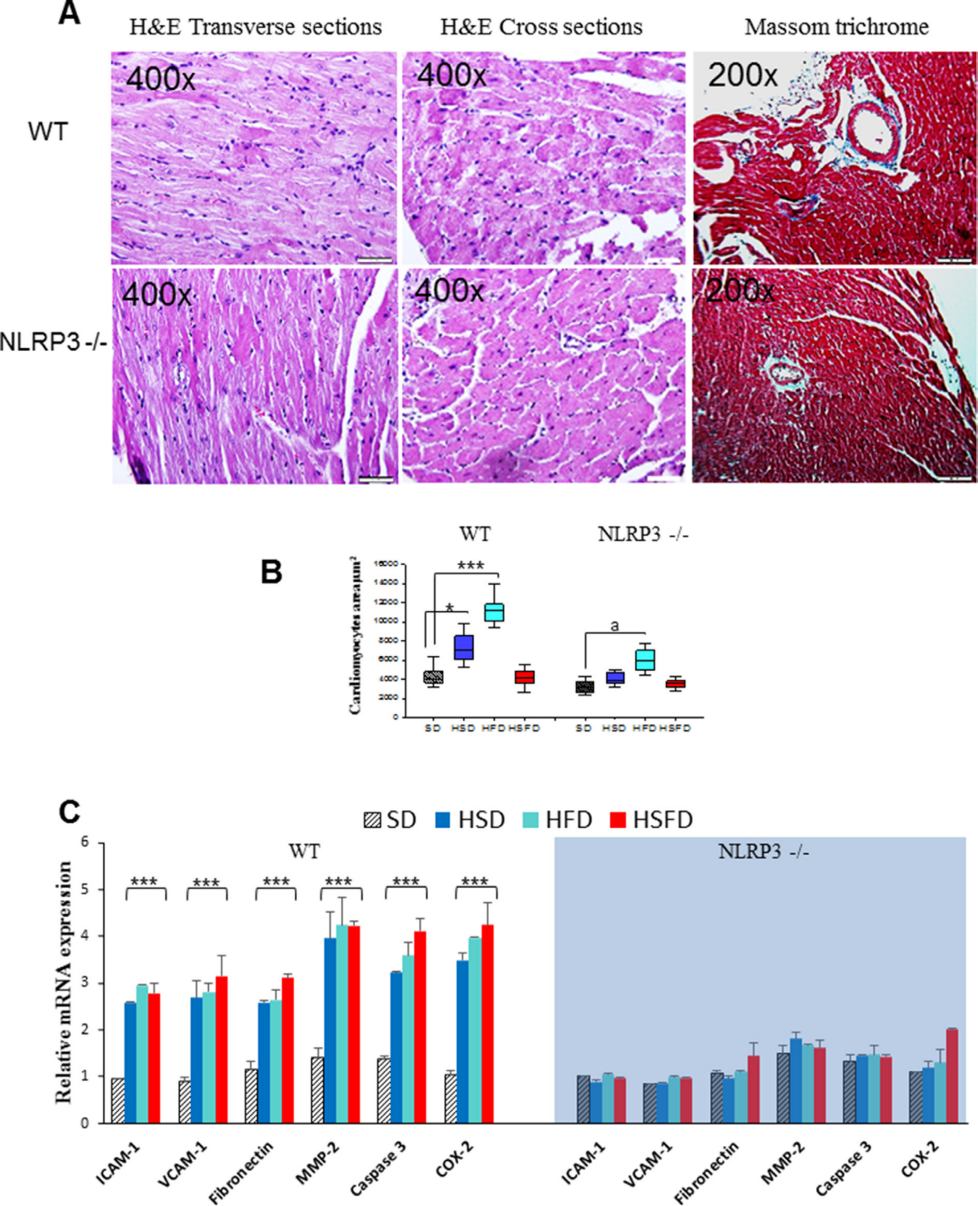
Histological analyses and gene expression changes of adhesion molecules and fibrosis markers in heart **(A)** Representative hematoxylin-and-eosin–stained micrographs showing transverse sections of Left Ventricular (LV) myocardium, cross-sectional area and representative Masson trichrome–stained micrographs showing perivascular sections of myocardium of HFD from WT and NLRP3 -/-. Complete diets see Supplementary figure 2. **(B)** Quantitative analysis of cardiomyocyte cross-sectional (transverse) area with measurements of ≈100 cardiomyocytes from 3 to 6 mice per group. ^*^P < 0.05, ^**^P < 0.005, ^***^P < 0.001 HSD, HFD and HSFD *vs* SD. **(C)** Relative gene expressions of ICAM-1 and VCAM-1 (mean±SEM) determined by quantitative PCR in heart from WT and NLRP3 -/-. Relative gene expressions of MMP-2, and fibronectyn and relative gene expressions of Caspase 3 and COX-2 determined by quantitative PCR in heart from WT and NLRP3 -/-. All data are presented as means ± SEM, n =6 mice; ^*^P < 0.05, ^**^P < 0.005, ^***^P < 0.001 HSD, HFD and HSFD *vs* SD.

To assess the impact of the diets on myocardial histology, cardiomyocyte cross-sectional area and fibrosis were examined. In the hematoxylin-and-eosin–stained sections, HSD and HFD but not HSFD showed a disarrangement of myofibrils and increased cardiomyocyte transverse cross-sectional area. However, NLRP3 -/- groups only showed a moderate increase in HFD (Figures 4A, 4B and Supplementary Figure 2). Further examination with Masson trichrome staining revealed overt perivascular fibrosis after diets in WT group without significant changes in NLRP3 -/- group (Figure 4A and Supplementary Figure 2). We also studied the changes in gene expression of several biomarkers of endothelial activation and fibrosis. Intercellular adhesion molecule 1 (ICAM-1) and vascular cell adhesion molecule 1 (VCAM-1) were increased in heart tissues of HSD, HFD and HSFD-fed animals, but no significant changes were observed in NLRP3 -/- animals exposed to the same diets (Figure 4D). Similarly, typical fibrosis biomarkers such as Matrix metallopeptidase 2 (MMP2) and fibronectin, were also increased in heart tissues of HSD, HFD and HSFD-fed WT animals but again, nonoteworthy changes were observed in NLRP3 -/- mice (Figure 4D). In the same line, apoptosis biomarker caspase 3 and endotelial inflammatory biomarker COX-2 were also increased in heart tissues of HSD, HFD and HSFD-fed animals but again, whilst no significative changes were observed in knockout animals (Figure 4D).

### Pharmacological inhibition of NLRP3 prevent of the HSD, HFD and HSFD-Induced obesity and heart damage

**Figure 5:**
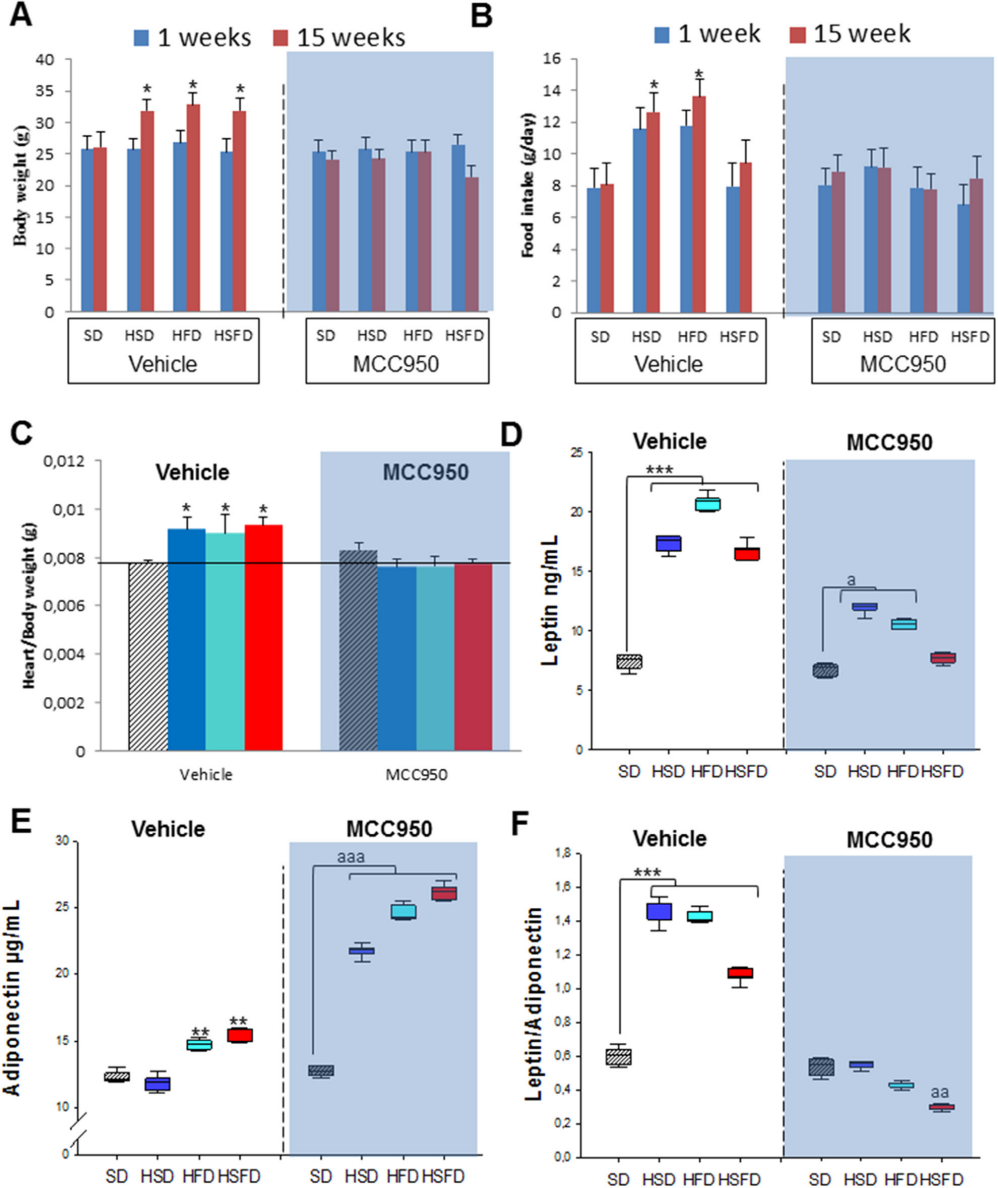
NLRP3 inhibition by MCC950 prevents obesity induced effects of the HSD, HFD and HSFD diets **(A)** Body weight chart of diets-induced obesity in WT-vehicle and MCC950 mice fed with HSD, HFD and HSFD diets. The mice were fed with the diets for 15 weeks and their body weights were monitored weekly. We represent the changes of the final week respect to the first week. **(B)** Average daily oral food intake normalized to body weight, measured on the different diets. The food intake of mice was measured was monitored weekly. We represent the changes of the final week respect to the first week. **(C)** Heart weight normalized to body weight. **(D-F)** Levels of leptin, adiponectin, and ratio in plasma. Blood samples were collected after overnight fasting. All data are presented as means ± SEM, n = 10 mice; ^*^P < 0.05, ^***^P < 0.005 HSD, HFD and HSFD *vs* SD; ^a^P < 0.05, ^aa^P < 0.005 MCC950 *vs* vehicle.

Using a pharmacological approximation, we have tested the effect of the same diets after inhibition of NLRP3 with MCC950. WT groups for this experiment were distributed in vehicle and the different diet+MCC950 (20 mg/Kg/day i.p.) animals. After fifteen weeks, all obesogenic diets led to increased weight in the vehicle group (Figure 5A), however the pharmacological inhibition of NLRP3- protected these animals from obesity (Figure 5A), similar to the genetic NLRP3 deletion. The daily food intake was lower in MCC950-treated groups compared to vehicle groups but, interestingly, with comparable relative changes among the different diets (Figure 5B). The different diets were shown to increase the heart weight in vehicle groups (Figure 5C and Supplementary Figure 3), with no change after MCC950 administration. Furthermore, MCC950 reduced cardiomyocytes area and perivascular fibrosis induced by diets (Supplementary Figure 4).

The metabolic studies performed with these groups of animal showed how the treatment with the MCC950 inhibitor ameliorated the effect on several circulating metabolites. Thus, the increased leptin and leptin/adiponectin ratio observed after HSD, HFD and HSFD feeding in vehicle groups, was reduced in the MCC950-treated groups with elevated adiponectin levels (Figures 5D-5F). Similarly, some alterations of biomarker levels in serum including glucose, cholesterol, triglycerides, hepatic transaminases, uric acid and creatinine (Supplementary Table 3) were reverted in the MCC950-treated mice.

**Figure 6:**
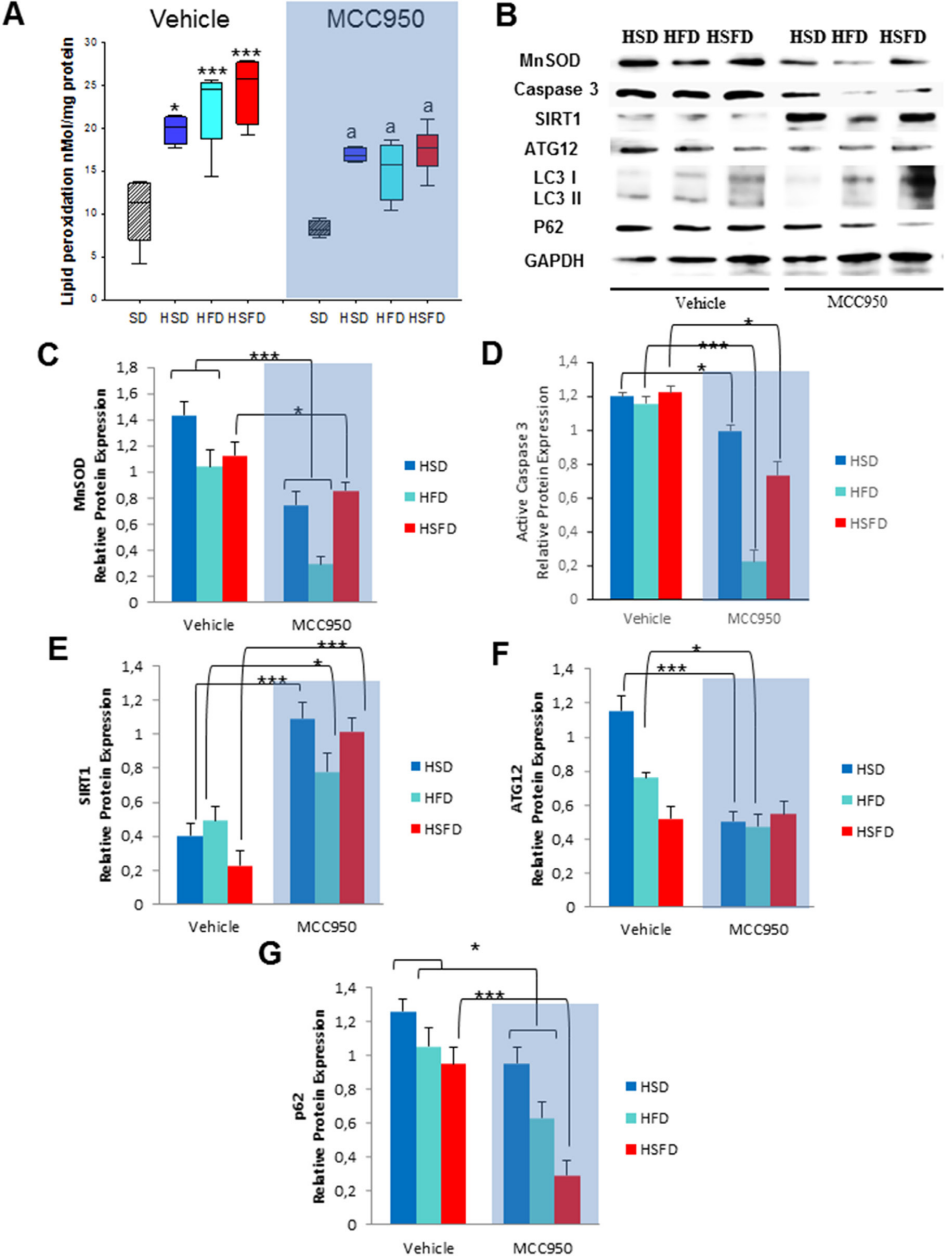
Changes in oxidative stress, apoptosis and autophagy in response to MCC950 **(A)** Lipid peroxidations. **(B)** Protein expression levels by western blot of MnSOD antioxidants in heart after MCC950 treatment in mice. We compare HSD, HFD and HSFD diets with MCC950 treated groups. **(C)** Densitometric quantification of protein expression levels of the antioxidant in heart tissues from mice relative to GAPDH (loading control). **(D)** Densitometric quantification of protein expression levels of the active Caspase 3 protein. **(E)** Densitometric quantification of protein expression levels of Sirt1 in heart from mice. **(F** and **G)** Densitometric quantification of protein expression levels of autophagy markers ATG12 and p62 in the heart in the hearts after diets and MCC950 treatment. All data are presented as means ± SEM with a representative blot, n= 4–5 mice; ^*^P < 0.05, ^***^P < 0.001 MCC950 *vs* vehicle.

Guided by these findings on several metabolic pathways, we examined the effect of the obesogenic diets on oxidative stress, apoptosis and autophagy in heart tissues and compared to the observations described with NLRP -/- mice. As expected, we observed that the inhibitor resulted in a significant reduction of lipid peroxidation compared to those in the HSD, HFD and HSFD-vehicle groups together with a reduction of MnSOD protein expression (Figures 6A-6C). Additionally, MCC950 also induced down-regulation of the active caspase 3 apoptotic protein (Figures 6B and 6D).

We also observed the same protective mechanisms in cardiac tissues determined by the high overexpression levels of SIRT1 in heart tissues of MCC950 treated groups (Figures 6B and 6E). Consistent with the NLRP3 -/- protective phenotype, we observed an increase of ATG12 and LC3II protein expression after diets with a p62/SQSTM1 accumulation, which were restored after treatment with MCC950 (Figures 6B, 6F and 6G). This autophagy induction was also demonstrated in an *in vitro* experiment. THP-1 cells treated with this inhibitor showed similar induction of autophagy as with rapamycin with an improved autophagic flux (Supplementary Figure 5). MCC950 also reduced serum biomarkers of inflammation such as IL-1β and TNF-α associated with the different diets (Supplementary Figure 6).

### Serum from HSD, HFD and HSFD-fed mice induce inflammasome and inflammation activation in monocytes

**Figure 7:**
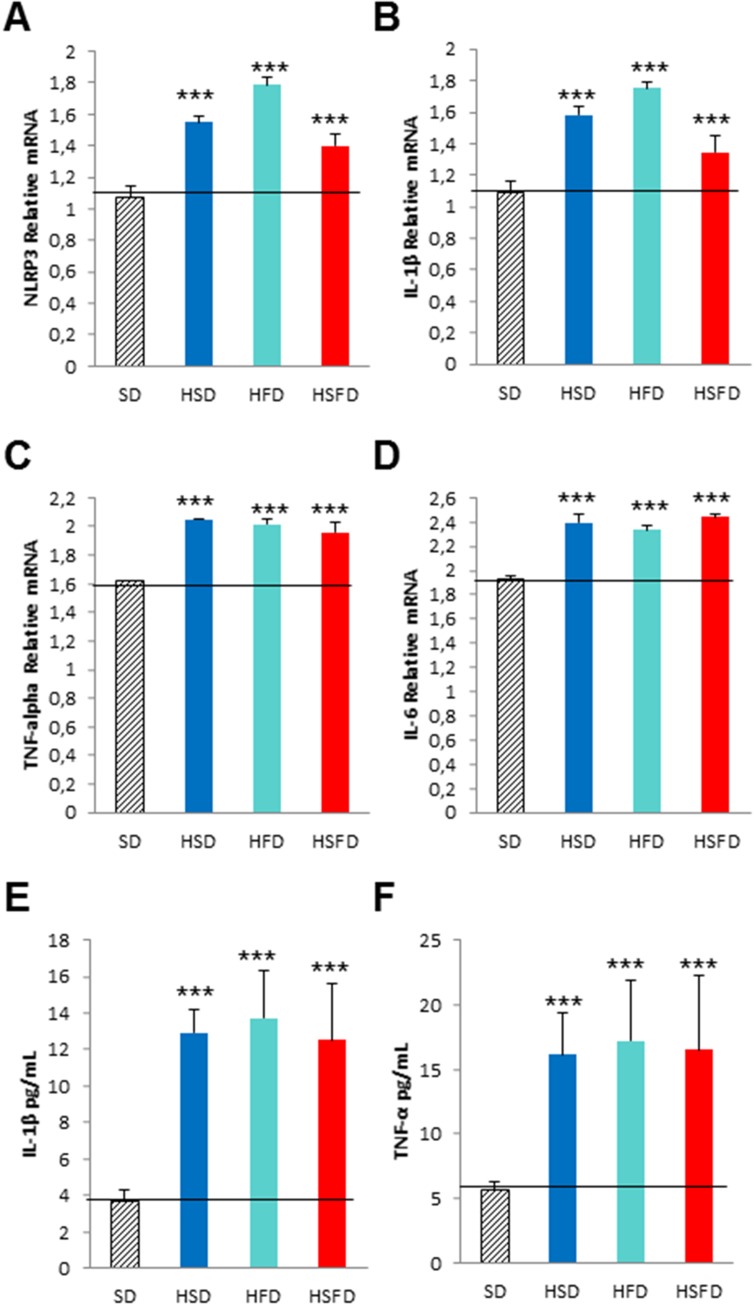
Pro-inflammasome and pro-inflammation effects in THP-1 monocytes cultivated with different sera from WT mice fed with HSD, HFD and HSFD diets **(A** and **B)** Relative gene expression of NRLP3 and IL-1β determined by quantitative PCR in THP-1 cells. **(C** and **D)** Relative gene expression of TNF-α and IL-6 determined by quantitative PCR in THP-1 cells. **(E** and **F)** IL-1β and TNF-α medium release from THP-1 cells. THP-1 monocytes was assessed after a 24 hr incubation with media containing serum from mice fed with SD, HSD, HFS, and HSFD diets for 15 weeks. The graphs show the mean ± SEM from three independent experiments. ^***^P < 0.01.

**Figure 8:**
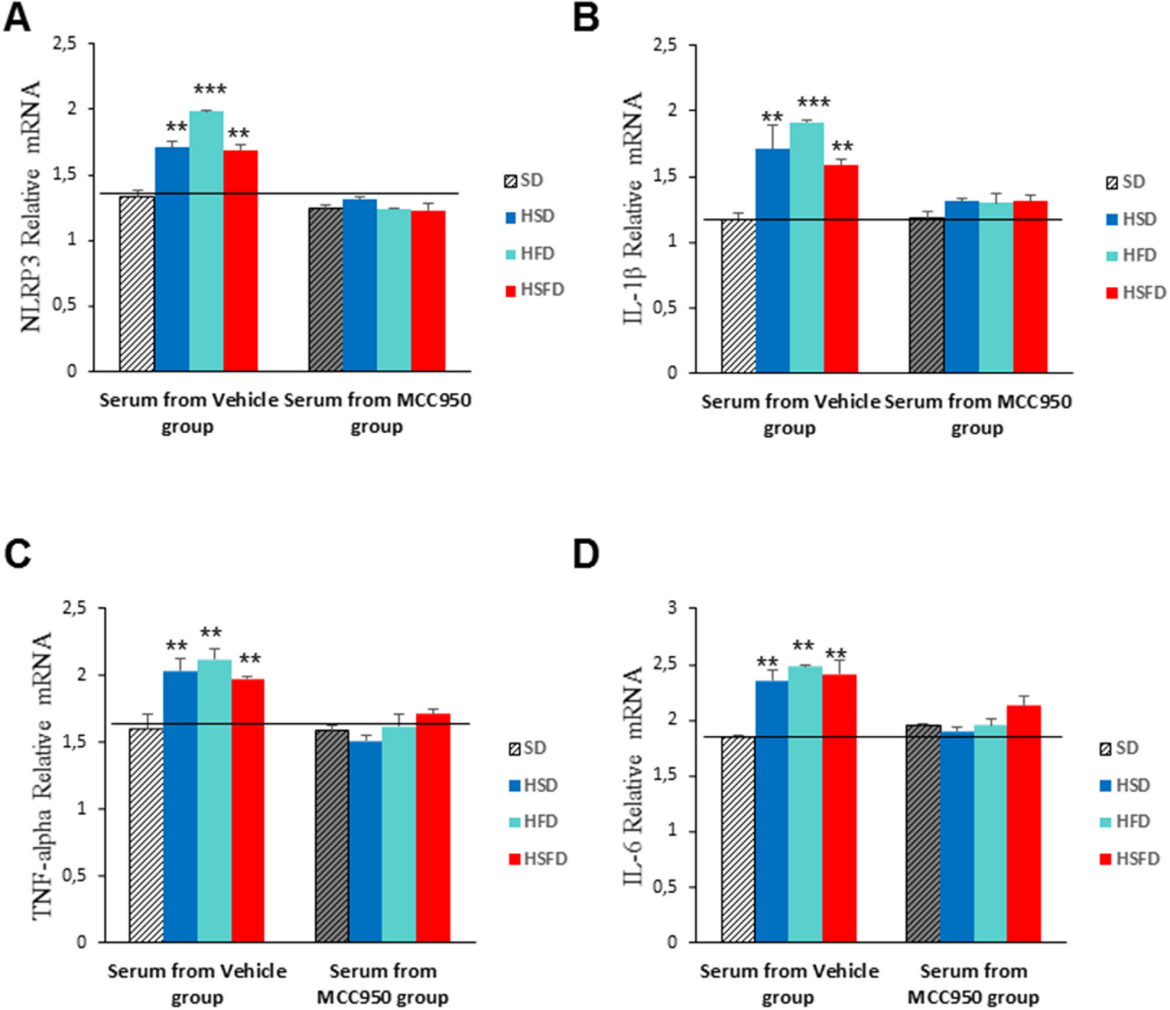
Inflammasome inhibition in THP-1 monocytes cultivated with sera from MCC950-treated WT mice fed with HSD, HFD and HSFD diets **(A** and **B)** Relative gene expression of NRLP3 and IL-1β determined by quantitative PCR in THP-1 cells. **(C** and **D)** Relative gene expression of TNF-α and IL-6 determined by quantitative PCR in THP-1 cells. THP-1 monocytes were assessed after a 24 hr incubation with media containing serum from mice fed with SD, HSD, HFS, and HSFD diets and mice fed with SD, HSD, HFS, and HSFD and treated with MCC950 for 15 weeks. The graphs show the mean ± SEM from three independent experiments. ^*^P < 0.05, ^*^P < 0.005, ^***^P < 0.001.

It is known that some soluble circulating factors can induce cardiac damage and systemic inflammation [19]. We next sought to investigate if serum separated from these mice are able to induce these alterations in cultured cells. Since the diets induced increased released levels of IL-1β and TNF-α in serum, an inflammatory phenotype could be induced in cells. THP-1 monocytes were incubated for 24 hr with media containing serum from mice fed with either SD, HSD, HFS, and HSFD diets for 15 weeks and the inflammatory profiles were determined. Our results indicate that incubation with these sera show an increase in inflammasome gene expression NLRP3 (p<0.01) and IL-1β (p<0.01) (Figure 7A and 7B) and other inflammatory genes such as TNF-α (p<0.01) and IL-6 (p<0.01) (Figures 7C and 7D, which were accompanied by IL-1β and TNF-α medium release (Figures 7E and 7F). Interestingly, the incubation with media containing different sera from mice fed with SD, HSD, HFS, and HSFD diets and treated with MCC950 showed a reduction in inflammasome (p<0.01) (Figures 8A and 8B) and other inflammatory genes expression (p<0.01) (Figures 8C and 8D). This may be due to the high protein binding of MCC950 (a sulfonyl urea), which results in a significant amount of drug in serum, being available for release in the *in vitro* assay.

## DISCUSSION

It has been shown that chronic over-nutrition: i) can induce inflammasome activation in several metabolic tissues such as adipose, renal and hepatic tissues [7–9], ii) can activate the NLRP3-inflammasome in macrophages [20], iii) has been involved in obesity-induced inflammation and insulin resistance [21], and iv) can initiate intestinal inflammation in epithelial cells [22]. In this context, activation of NLRP3 inflammasome mediating IL-1β and IL-18 secretion has emerged as an important component of inflammatory processes in cardiovascular diseases [23, 24]. Our findings provide interesting data of how these obesogenic diets promote the activation of NLRP3-inflammasome in the heart. The HSD, HFD and HSFD feeding induced not only an important increment in body and heart weights, but also an increment in several important cardiovascular risk biomarkers such as high levels of leptin and leption/adiponectin ratio [25] as well as alterations in several biochemical parameters and tissue damage biomarkers. These diets also induced cardiac damage by increased oxidative stress, inflammation, apoptosis and an impaired autophagy process. Corroborating existing literature, all these physical and metabolic alterations have been observed in HFD and HSD, and both have been closely linked to an increased obesityrisk, cardiac disease, diabetes (T2D), metabolic syndrome, non-alcoholic fatty liver disease [26].

Autophagy is a critical mechanism to preserve cellular homeostasis and has been implicated in the maintenance of cardiac structure and function under physiological and/or pathological conditions. Different studies have demonstrated an indispensable role of this process in the etiology of cardiac anomalies under obesity and metabolic syndrome [27]. In fact, obesity induced by HFD showed an impairedautophagic flux in heart tissue [28] similar to our observations. A high-carbohydrate (fructose) has also been shown to induce the same modifications in hepatic tissues [29], which could corroborate our observations in heart induced by all high caloric diets. Obesity is recognized as a chronic, low-grade, systemic inflammation, which contributes to the development of several metabolic and cardiovascular diseases [4]. The fact that these observed effects were partially reproduced in THP-1 monocytes incubated with media supplemented with serum from mice fed HSD, HFS or HSFD suggest systemic effects of high glucose, cholesterol or other serum factors with potential interest to the development of these metabolic and inflammatory adaptation, and autophagic impairment.

The role of NLRP3-inflammasome in the HFD-induced and obesity-related cardiovascular disease has been poorly studied. However, NLRP3 in subcutaneous adipose tissue has been associated with the severity of coronary atherosclerosis [30]. A recent study has shown genetic variants of NLRP3 in the pathogenesis of atherosclerosis [31], and NLRP3 and inflammatory cytokines have also been proposed as new cardiovascular risk biomarkers [32]. Taken together, these data suggest that the inhibition of inflammasome complex could help to protect the progression of many metabolic and cardiovascular diseases. Our results demonstrate that the NLRP3 deficient mice are protected from HSD, HFD and HSFD-associated body-weight gain, left ventricle hypertrophy and increased myocardial mass and related cardiomyopathies, and the disappearance of different cardiac remodeling-related dysfunctions and the absence of alterations in key biochemical biomarkers. All these observations are in concordance with previous observations on the protective effect of inflammasome complex in mice and reducing HFD pathological effects [7-9, 20, 21]. However, similarly to previous studies [7, 33], increased leptin levels in NLRP3 deficient mice was similar to WT mice on HSD and HFD, whereas NLRP3 knockout mice did not show changes in cardioprotective adiponectin levels [34]. NLRP3 deletion also showed a moderate but not significant oxidative stress increase, which was not enough to induce antioxidants synthesis in these animals. These results were accompanied by increased levels of pro-inflammatory TNF-alpha but not the IL-1β in WT animals in obesogenic diets. However, NLRP3 -/- mice showed attenuated increases in pro-inflammatory mediators, strengthening the idea that these knockout animals are more protected from damages induced by these diets. These observations were accompanied by reduced apoptosis induction in NLRP3 -/- mice with increased expression of the anti-apoptotic Bcl-2 and Sirt1, a NAD+-dependent class III histone deacetylase, which has been shown to protect from endothelial dysfunction, atherothrombosis, diet-induced obesity, type 2 diabetes, liver steatosis, and myocardial infarction [35]. In this sense, NLRP3 -/- mice showed no modifications in gene expression of potential vascular dysfunction and regulators of fibrosis induced in heart tissues by the diets. Interestingly, very recent and elegant studies have shown that mice deficient in inflammasome components are resistant to endothelial dysfunction and fibrosis induced by different challenges in liver, lung or kidney, similar to our observations in the heart [36–38]. In mammalian cells, Sirt1 modulate distinct metabolic and stress-response pathways including autophagy [39] so, the increased expression of this protein in NLRP3 -/- mice could improve the capacity for metabolic adaptation and/or cardiovascular protection. Likewise, NLRP3 -/- mice showed a very efficient autophagy flux. Collectively, all these studies and the recent findings that these mice have also efficient autophagy flux in lung tissues and improved resistance to hyperoxia [40] indicate that NLRP3 deficiency remarkably protect different organs from stress situations, such as the obesogenic diets.

Further, the inhibition of inflammasome activation recently been shown to induce an important protective effect in cardiac injury. Abderrazak et al., (2016) showed an inbihition of NLRP3-inflammasome and autophagy induction in ApoE2. Ki mice in high-fat diet after treatment with a natural product called arglabin [41]. Other novel inhibitor named as 16673-34-0, an intermediate substrate of the synthesis of glyburide free of the cyclohexylurea moiety, has a potent inhibitory effect limiting the secondary inflammatory response and reducing infarct size in a mouse model of acute myocardial ischemia-reperfusion injury [42]. More recently, MCC950 has been shown to reduce the infarct size and preserves cardiac function in a randomized, blinded translational large animal myocardial infarction model [43]. In our study, we have studied for the first time the protective effect of the same selective inhibitor, on cardiac tissues induced by obesogenic diets. We showed that daily intraperitoneal administration of MCC950 prevent from HSD, HFD and HSFD-associated body-weight gain, heart-weight gain, increased circulating adiponectin, and maintained leptin and leptin/adiponectin levels, in line with this protection role. Our study also reveals that other circulating markers of tissue damage and inflammation were reduced in animals treated with MCC950. Furthermore, results in MCC950 treated mice reproduced previous observations with NLRP3 -/- mice under oxidative stress displayedreduced apoptosis and induced Sirt1 expression accompanied by an improved autophagy flux in heart tissues. Finally, previous *in vitro* studies have shown a reduction of inflammation in cells treated with serum from HFD+Resveratrol fed animals because of anti-inflammatory effect and overexpression of Sirt1 induced by this compound [13, 44]. In line with these studies, we observed that THP-1 monocytes treated with media supplemented with serum from mice fed with HSD, HFD or HSFD+MCC950 diets had lower mRNA expression of the inflammasome (NLRP3 and IL-1β) and inflammation (TNF-α and IL-6) components when compared to the chronic overfeeding animals. Taken together, these data indicate that monocytes could contribute, at least in part, to the beneficial effects of MCC950 in systemic inflammation.

In conclusion, our data suggests that the inhibition of NLRP3-inflammasome produces beneficial metabolic, inflammatory and could induce an autophagic adaptations in the heart of diet-induced obesity mice models. In addition, it is possible that monocytes could participate in the heart damage induced by systemic inflammation after chronic exposure to unhealthy diets. Although lifestyle changes are the primary interventions to prevent obesity and consequent heart failures associated to obesogenic diets, the damages occasioned in cardiac tissues after prolonged unhealthy habits can be irreversible. In these cases, the use of inflammasome inhibitors such as MCC950, with a potential autophagic adaptation of the tissues, may hold a promise as therapy to ameliorate heart damage and metabolic changes, which accelerates CVD.

## MATERIALS AND METHODS

### Ethical statements

Animal studies were performed in accordance with the European Union guidelines (2010/63/EU) and the corresponding Spanish regulations for the use of laboratory animals in chronic experiments (RD 53/2013 on the care of experimental animals). All experiments were approved by the local institutional animal care committee.

### Animals

For all experiments, only male mice were used. C57/BL6/J mice and NLRP3−/− transgenic mice (C57BL/6J background), weighing 25-30 g were maintained on a regular 12 h light/dark cycle. Diets were started at 16 weeks of age after randomization into four groups. These groups correspond to the following dietary regimens: i) regular chow or standard diet (SD) from Teklad Global 14% Protein Rodent Maintenance Diet, Harlan Laboratories (carbohydrate:protein:fat ratio of 48:14:4 percent of kcal); ii) a HFD consisting of Teklad Global modified to provide 45% of calories from fat; iii) a high sugar diet consisting of Teklad Global modified to provide 32% of calories from sucrose and a HSFD with the combination of fat and sucrose. MCC950 was included in different groups with the same diets in daily doses of 20 mg/kg (drug/kg body weight). All groups had ad libitum access to their prescribed diet and water throughout the study. Body weight and food intake were monitored weekly. Animal rooms were maintained at 20–22°C with 30–70% relative.

### Reagents

Monoclonal antibodies specific for Beclin-1 and p62 were purchased from Sigma-Aldrich (Saint Louis, USA). Anti-GAPDH monoclonal antibody was acquired from Calbiochem-Merck Chemicals Ltd. (Nottingham, UK). Similarly, anti-NLRP3 antibody was purchased from Adipogen (San Diego, USA); while anti-active caspase-3, anti-SIRT-1 and anti-Parkin were obtained from Cell Signalling Technology (Beverly, MA, USA). Finally, anti-IL-1β (p17), anti-OGG-1, anti-Bcl-2, anti-Bax, anti-MnSOD, anti-catalase, anti-ATG12 and anti-MAP-LC3 antibodies from (Santa Cruz Biotechnology). A cocktail of protease inhibitors (Complete™ Protease Inhibitor Cocktail) was purchased from Boehringer Mannheim (Indianapolis, IN). The Immun Star HRP substrate kit was obtained from Bio-Rad Laboratories Inc. (Hercules, CA).

### *In vitro* cell experiments

THP-1 cells, a human leukemia monocytic cell line extensively used to study monocyte/macrophage functions, were cultured at 37°C in a 5% CO_2_ atmosphere in RPMI-1640 medium supplemented with L-glutamine, an antibiotic/antimycotic solution (Sigma Chemical Co., St. Louis, MO, USA), and 10% fetal bovine serum. THP-1 cells were incubated for 24 hr with media containing serum from mice under SD, HSD, HFD and HSFD diets.

### IL-1β and TNF-α levels

Serum or culture medium levels of IL-1β (GenWay, San Diego CA, USA) and TNF-α (Biosource, UK, and GenWay, San Diego, USA) were assayed in duplicate using commercial ELISA kits.

### Leptin and adiponectin

Serum levels of leptin and adiponectin were assayed in duplicate using commercial ELISA kits (R&D Systems, Minneapolis, USA).

### Serum biomarkers

Serum levels of glucose, triglycerides, cholesterol, uric acid, aspartate aminotransferase, alanine aminotransferase and creatine kinase were assayed using commercial kits (Randox Laboratories, Antrim, UK).

### Immunoblotting

Western blotting was performed using standard methods. After protein transfer, the membrane was incubated with various primary antibodies diluted 1:1000, and then with the corresponding secondary antibody coupled to horseradish peroxidase at a 1:10000 dilution. Specific protein complexes were identified using the Immun Star HRP substrate kit (Biorad Laboratories Inc., Hercules, CA, USA).

### Lipid hydroperoxides

The FOX assay was carried out according to the method previously reported [45]. The FOX reagent was prepared by mixing in the order: 90 ml methanol, 88 mg BHT, 10 ml 250 mM H_2_SO_4_, 9.8 mg ammonium ferrous sulfate hexahydrate and 7.6 mg xylenol Orange. 680 μl of FOX reagent were added to 320 μl of each sample, and the solution was incubated for 30 min at 37 °C with gentle shaking. After a short high-speed centrifugation (3,000×g for 1 min at room temperature), samples absorbance was read at 560 nm against the blank (0.9% NaCl and FOX reagent). For hydroperoxides quantification, a serial standard dilution of hydrogen peroxide was used.

### Protein carbonyl

Protein carbonyls were assayed in duplicates by commercial ELISA kits using a commercial kit from Cayman Chemical (Ann Arbor, Michigan, USA).

### Histological study

After anesthesia of mice, hearts were excised and immediately placed in a 10% neutral-buffered formalin at room temperature for 24 hours after a brief rinse with PBS. The specimens were embedded in paraffin, cut in 5-μm sections, and stained with hematoxylin and eosin. Cardiomyocyte cross-sectional areas were calculated on a digital microscope (×400) with ImageJ (version 1.34S) software. Masson’s trichrome staining was used to detect fibrosis in heart sections and fibrotic areas were also calculated on a digital microscope (×400) with ImageJ (version 1.34S) software.

### Real-time quantitative PCR

The expression of NLRP3 gene was analyzed by SYBR Green quantitative PCR of mRNA extracted from cardiac tissues. Total cellular RNA was purified from the cells using the Trisure method (Bioline, London, UK). RNA concentration was determined spectrophotometrically. Contaminating genomic DNA was removed by incubation of one microgram of total RNA from each sample with gDNA wipeout buffer (Quantitect Reverse Transcription Kit, Qiagen. Hilden, Germany) for 5 min at 42°C. RNA samples were subsequently retrotranscribed to cDNA using the QuantiTect Reverse Transcription Kit (Qiagen. Hilden, Germany). Thermal cycling conditions used were denaturation at 95°C for 20 s, 40 cycles of priming at at 54°C for 20 s, and elongation at 72°C for 20 s. Primers used can be consulted in Supplementary Table 1. All reactions were performed in duplicate. Reaction mixtures, without RNA, were used as negative controls in each run. Absence of genomic DNA contamination was confirmed by setting up control reactions that do not contain reverse transcriptase. Fold changes in the expression of genes of interest were calculated using the ΔΔCt method.

### Statistics

All data are expressed as means ± SEM. After, evaluation of normality using Shapiro-Wilk test, statistical differences among the different groups were measured using either an unpaired Student t test or 1-way analysis of variance (ANOVA) when appropriate with Tukeys post-hoc test. A P value of ≤0.05 was considered statistically significant. Statistical analyses were performed using Prism software version 5.0a (GraphPad, San Diego, CA). Asterisks in the figures represent the following: ^*^: P ≤0.05; ^**^: P ≤ 0.01; and ^***^: P ≤ 0.001.

## SUPPLEMENTARY MATERIALS FIGURES AND TABLES



## Abbreviations

8-oxoG: 7,8-dihydro-8-oxoguanine
CVD: Cardiovascular disease
HFD: High fat diet
HSD: High sugar diet
HSFD: High sugar and fat diet
IL-(1β,18): interleukins
NLRP3: NOD-like receptor family, pyrin domain containing 3
OGG-1: 8-oxoguanine glycosylase
