# Supplementary material for: NLRP3-inflammasome inhibition prevents high fat and high sugar diets-induced heart damage through autophagy induction
Source: Oncotarget. 2017 Sep 8;8(59):99740–56. doi: 10.18632/oncotarget.20763 (PMC5725128; doi:10.18632/oncotarget.20763)
Supplement: Supplementary file 1 [file oncotarget-08-99740-s001.pdf]

NLRP3-inflammasome inhibition prevents high fat and high sugar diets-induced heart damage through autophagy induction

SUPPLEMENTARY MATERIALS

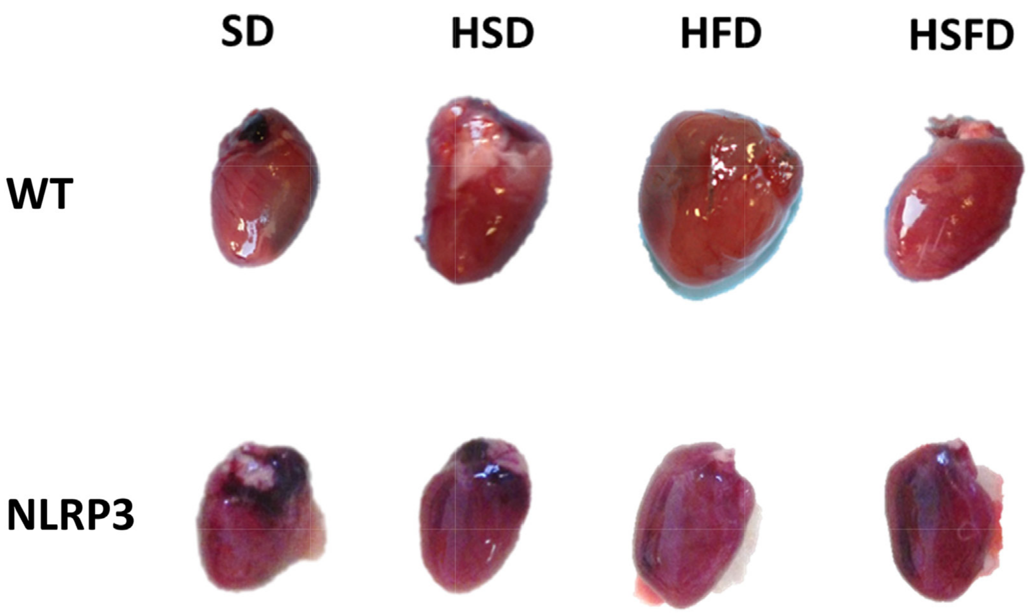

Supplementary Figure 1: Representative pictures of heart from WT and NLRP3 -/- mice fed with standard diet and High Sugar Diet (HSD), High Fat Diet (HFD) and High Sugar-Fat Diet (HSFD).

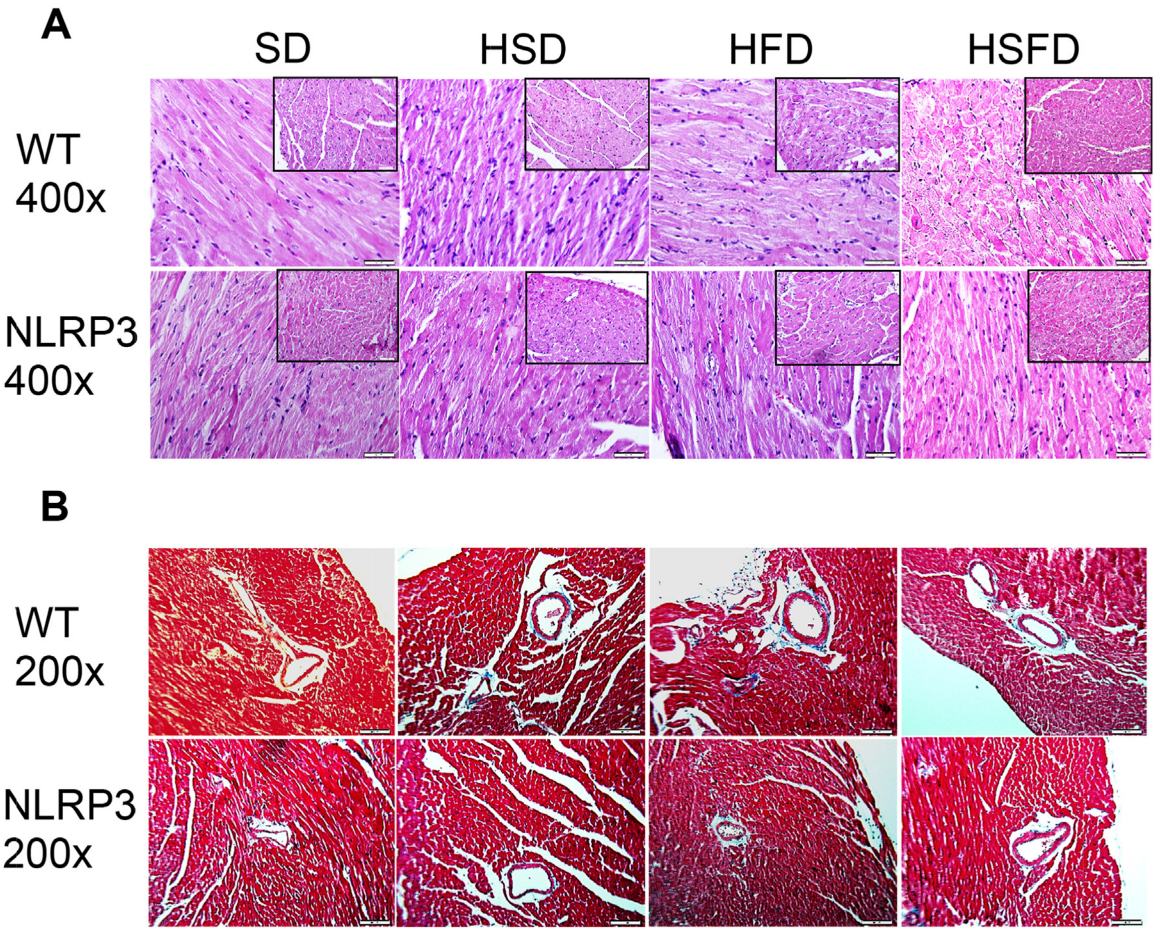

Supplementary Figure 2: Histological analyses. **(A)** Representative hematoxylin-and-eosin–stained micrographs showing transverse sections of Left Ventricular (LV) myocardium. Top right insets show representative cross-sectional area **(B)** Representative Masson trichrome–stained micrographs showing perivascular sections of myocardium.

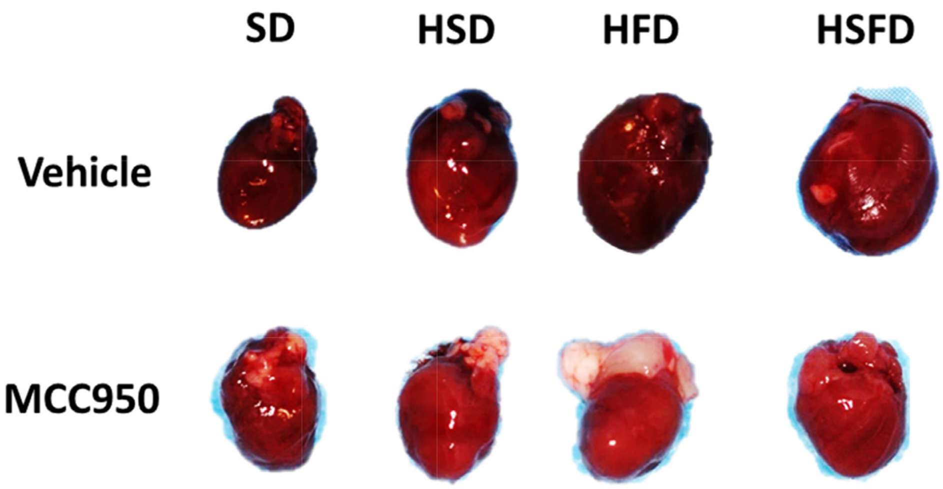

Supplementary Figure 3: Representative pictures of heart from mice fed with standard diet and High Sugar Diet (HSD), High Fat Diet (HFD) and High Sugar-Fat Diet (HSFD) and treated with vehicle or the NLRP3 inhibitor MCC950.

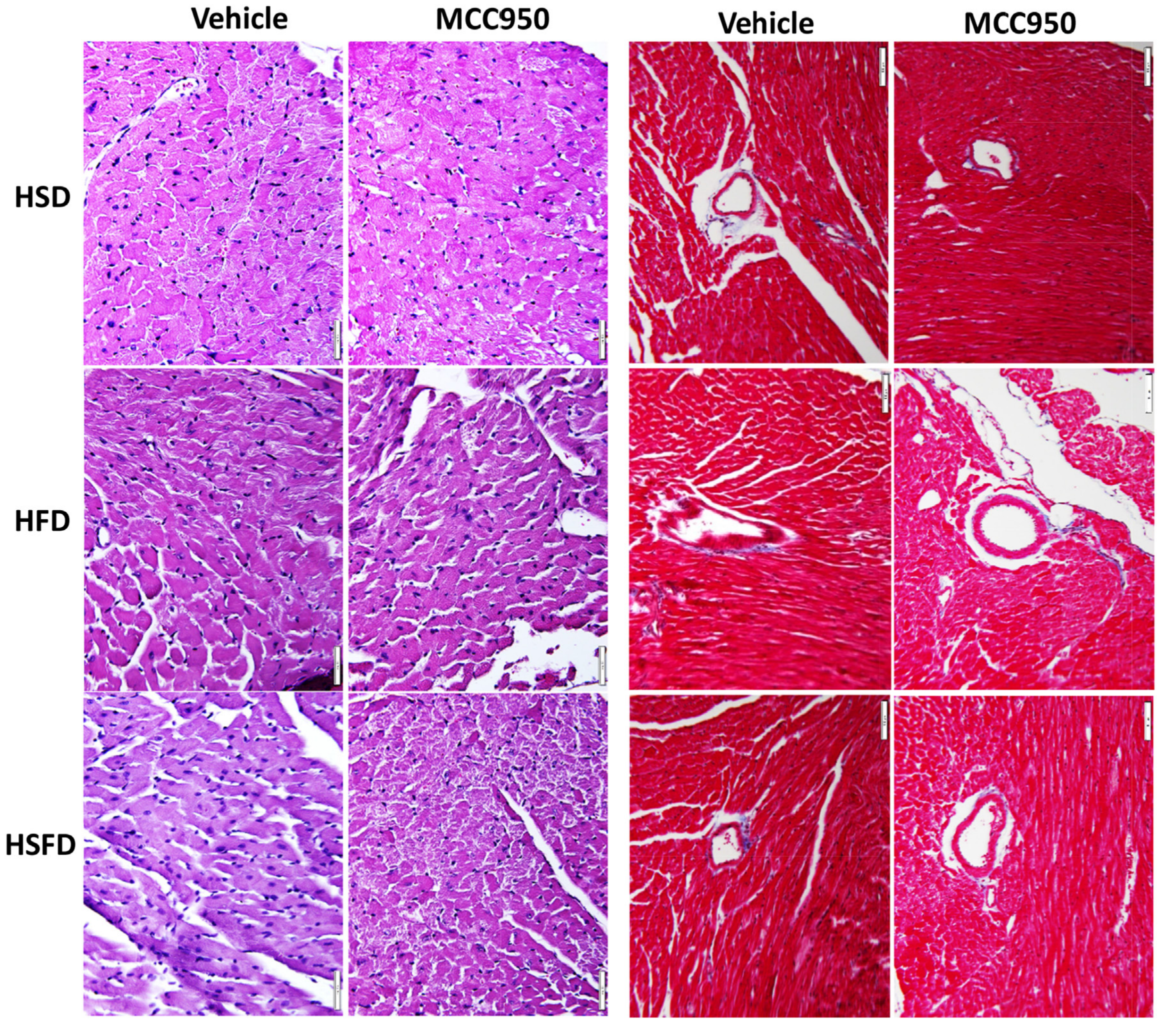

Supplementary Figure 4: Histological analyses. Representative hematoxylin-and-eosin–stained micrographs showing transverse sections of Left Ventricular (LV) myocardium and representative Masson trichrome–stained micrographs showing perivascular sections of myocardium.

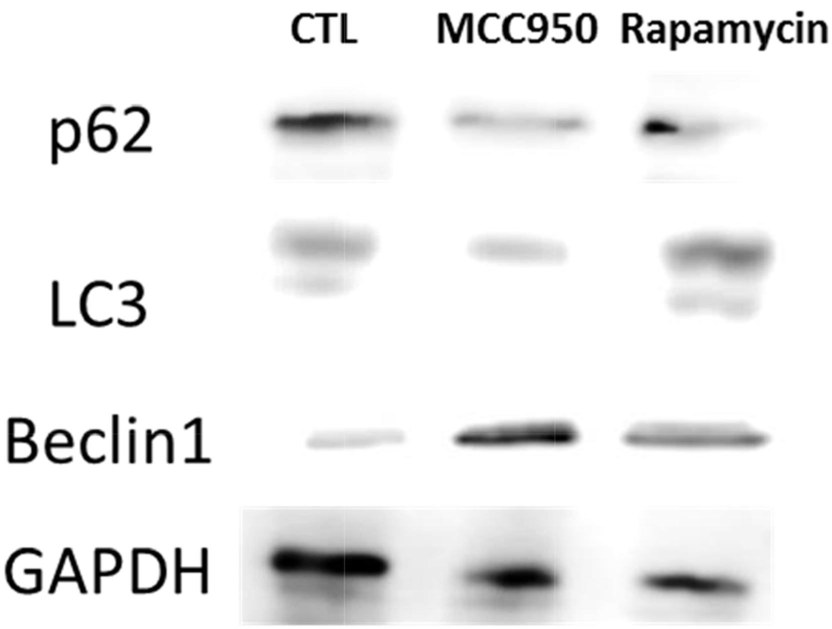

Supplementary Figure 5: Comparative effect of MCC950 and rapamycin in the autophagy induction in an *in vitro* assay in THP-1 cells. Western blot analysis showing autophagy markers p62/SQSTM1 accumulation, LC3 and Beclin 1 in the THP-1 cells.

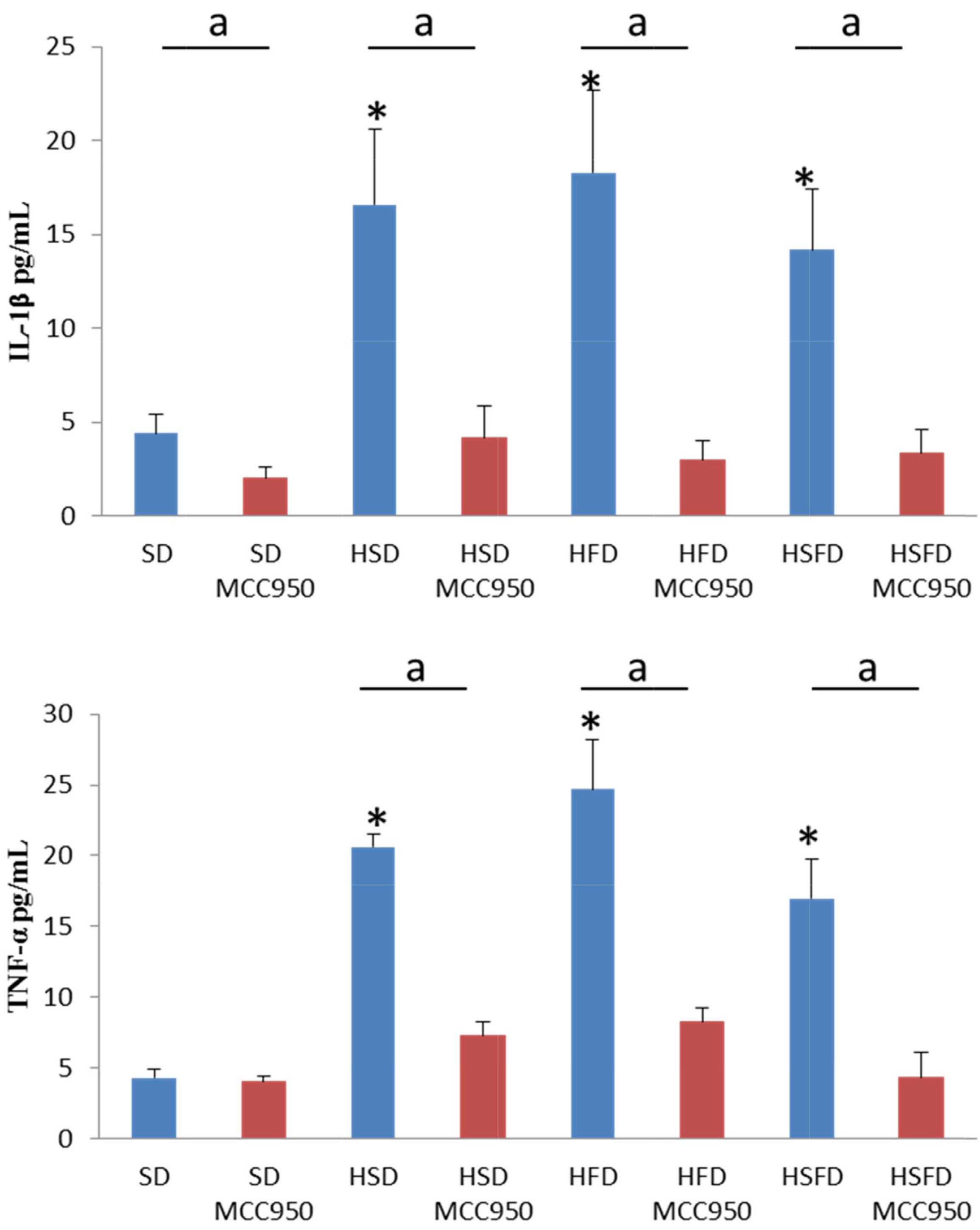

Supplementary Figure 6: IL-1β and TNF-α levels in serum from diets-induced obesity in WT-vehicle and MCC950 mice fed with HSD, HFD and HSFD diets were determined by ELISA. All data are presented as means ± SEM with a representative blot, n = 10 mice; *P < 0.05 *vs* SD and ^{a}P < 0.05 comparing vehicle *vs* MCC950.

Supplementary Table 1: Primers of the gene expression study

Gen amplificado	Secuencia
VCAM-1	Primer forward: TTATTGTTGACATCTCCCCCG<br>Primer reverse: TCATTCCTTACCACCCCATTG
ICAM-1	Primer forward: AACTTTTCAGCTCCGGTCCTG<br>Primer reverse: TCAGTGTGAATTGGACCTGCG
MMP2	Primer forward: CAAGGACCGGTTTATTTGGC<br>Primer reverse: ATTCCCTGCGAAGAACACAG
Fibronectina	Primer forward: GCAGTGACCAACATTGATCGC<br>Primer reverse: AAAAGCTCCCGGATTCCATCC
Caspase 3	Primer forward: TGGACTGTGGCATTGAGACAG<br>Primer reverse: CGACCCGTCCTTTGAATTTC
COX-2	Primer forward: GTGTATCCCCCCACAGTCAAA<br>Primer reverse: ACACTCTGTTGTGCTCCCGAA
TNF-α	Primer forward: CTCAACTGGTGTCGAGAAGTCC<br>Primer reverse: TTCCTTGAGCGTGCTGAACAGC
IL-6	Primer forward: AGACAGCCACTCACCTCTTCAG<br>Primer reverse: TTCTGCCAGTGCCTCTTTGCTG
NLRP3	Primer forward: GGAGAGACCTTTATGAGAAAGCAA<br>Primer reverse: GCTGTCTTCCTGGCATATCACA
IL-1β	Primer forward: TTACAGTGGCAATGAGGATGAC<br>Primer reverse: GTCGGAGATTCGTAGCTGGAT

Supplementary Table 2: Effects of the diets in *wild-type* and NLRP3 -/- mice on various biomarkers in plasma

Parameters	*Wild-type*				NLRP3			
	SD	HSD	HFD	HSFD	SD	HSD	HFD	HSFD
Cholesterol (mg/dL)	304.9 (10.5)	267.7 (16.7)	364.5 (10.9)*	395 (12.3)*	285.2 (8.4)	263 (9.2)	300.1 (10.8)†	323.2 (11.4)†
Triglycerides (mg/dL)	37.84 (7.3)	32.29 (6.3)	34.02 (7.1)	31.94 (5.2)	48.61 (10.3)	70.13 (11.1)	66.66 (12.3)	29.38 (8.8)
Glucose (mg/dL)	151.4 (10.5)	195.4 (11.3)*	172.9 (10.6)	183.9 (10.8)*	117.5 (12.4)†	126.3 (10.1)†	104 (9.6)†	109.6 (9.4)†
Albumin (mg/dL)	2.31 (0.12)	2.02 (0.11)	3.11 (0.11)*	1.72 (0.14)	1.93 (0.10)	2.08 (0.12)	2.05 (0.13)	2.04 (0.14)
Bilirubin (mg/dL)	0.16 (0.01)	0.14 (0.02)	0.17 (0.03)	0.15 (0.02)	0.18 (0.03)	0.15 (0.02)	0.19 (0.03)	0.18 (0.02)
Ala aminotransferase (UL)	320.8 (97)	670.83 (105)*	637.16 (115)*	414.16 (98)*	344.15 (91)	402.5 (99)†	431.66 (113)†	309.13 (110)†
Asp aminotransferase (UL)	321.1 (101)	576.3 (61)*	598.5 (75)*	364.8 (99)	291.3 (91)	269.5 (101)†	333.3 (112)†	350 (101)
Lactate dehydrogenase (UL)	1480 (215)	1121 (283)	4599 (393)*	2667 (205)*	1325 (198)	915 (101)†	1218 (196)†	1106 (151)†
Creatine phosphokinase (UL)	3436 (723)	4815 (675)	5714 (745)*	6063 (697)*	3547 (845)	3857 (431)	4222 (250)†	4785 (640)†
Uric Acid (µMol/dL)	24.24 (2.3)	28.29 (1.3)*	32.33 (2.1)*	41.08 (1.9)	23.25 (1.5)	24.92 (2.1)	26.94 (1.9)†	25.59 (1.8)†
Creatinine (mg/dL)	0.64 (0.01)	2.73 (0.51)*	1.92 (0.43)*	0.96 (0.03)*	0.80 (0.05)	1.24 (0.23)†	1.12 (0.28)	0.96 (0.04)

Values are presented as mean ± SEM. UL, units per litre. * $P<0.05$ versus standard diet. † $P<0.05$ versus same diet between WT and NLRP3. (n=10).

Supplementary Table 3: Effects of the diets and MCC950 on various biomarkers in serum

Parameters	Vehicle				MCC950			
	SD	HSD	HFD	HSFD	SD	HSD	HFD	HSFD
Cholesterol (mg/dL)	330.7 (18.5)	278 (13.2)	570.4 (25.1)*	430 (22)*	318.2 (6.9)	310 (10.1)†	370 (15.1)†	340 (15)†
Triglycerides (mg/dL)	38.2 (6.4)	43.1 (5.4)	33.5 (5.9)	38.1 (6.8)	35.6 (7.2)	37.9 (8.8)	25.9 (6.3)	28.9 (8.1)
Glucose (mg/dL)	158.1 (11.2)	203 (13.1)*	192.1 (10.1)*	195.2 (11)*	149.1 (10.6)	169.2 (11.3)†	128.8 (8.2)†	130.5 (13.6)†
Albumin (mg/dL)	2.8 (0.15)	2.7 (0.13)	3.9 (0.19)*	2.3 (0.18)	2.7 (0.13)	3 (0.11)	1.5 (0.15)†	2.9 (0.10)
Bilirubin (mg/dL)	0.17 (0.02)	0.16 (0.02)	0.16 (0.04)	0.14 (0.08)	0.16 (0.04)	0.17 (0.03)	0.17 (0.06)	0.17 (0.05)
Ala aminotransferase (UL)	350.1 (67)	560.3 (72)*	670.21 (95)*	690.2 (112)*	338.5 (67)	392.5 (109)†	371.1 (101)†	465 (120)†
Asp aminotransferase (UL)	342.3 (92)	546.8 (101)*	618 (111)*	664.1 (109)*	333.5 (78)	367.8 (71)†	373.8 (94)†	370 (108)†
Lactate dehydrogenase (UL)	1390 (113)	1229 (303)	4224 (193)*	3018 (251)*	1215 (165)	1121 (150)	1432 (167)†	1615 (174)†
Creatine phosphokinase (UL)	3513 (601)	4468 (521)	5901 (851)*	6111 (891)*	3603 (528)	3807 (541)	4012 (321)†	4400 (721)†
Uric Acid (µMol/dL)	20.6 (3.3)	29.9 (2)*	45.4 (1.8)*	47.3 (2.9)*	21.4 (2.1)	20.9 (2.2)†	23.8 (2.8)†	24.5 (2.6)†
Creatinine (mg/dL)	0.57 (0.03)	2.55 (0.42)*	1.99 (0.29)*	1.03 (0.05)*	0.65 (0.05)	1.03 (0.188)†	1.01 (0.11)†	0.89 (0.03)†

Values are presented as mean ± SEM. UL, units per litre. * $P<0.05$ versus standard diet. † $P<0.05$ versus same diet between vehicle and MCC950. (n=8).
